# Compulsive sexual behavior and paraphilic interests in adults with chronic tic disorders and Tourette syndrome: a survey-based study

**DOI:** 10.1038/s41443-023-00729-x

**Published:** 2023-07-19

**Authors:** Lille Kurvits, Safiye Tozdan, Tina Mainka, Alexander Münchau, Kirsten R. Müller-Vahl, Andrea E. Cavanna, Peer Briken, Christos Ganos

**Affiliations:** 1 Department of Neurology with Experimental Neurology, Charité University Medicine, Berlin, Germany; 2 Department of Neurology, University Medical Center Göttingen, Göttingen, Germany; 3 Institute for Sex Research, Sexual Medicine and Forensic Psychiatry, University Medical Center Hamburg-Eppendorf, Hamburg, Germany; 4 Berlin Institute of Health at Charité – Universitätsmedizin Berlin, BIH Biomedical Innovation Academy, BIH Charité Clinician Scientist Program, Berlin, Germany; 5 Institute of Systems Motor Science, University Medical Center Schleswig-Holstein, Campus Lübeck, Lübeck, Germany; 6 Department of Psychiatry, Social Psychiatry and Psychotherapy, Hannover Medical School, Hannover, Germany; 7 Department of Neuropsychiatry, Birmingham and Solihull Mental Health NHS Foundation Trust and University of Birmingham, Birmingham, UK; 8 School of Life and Health Sciences, Aston University, Birmingham, UK; 9 University College London and Institute of Neurology, London, UK; 10 Department of Child Neuropsychiatry, University of Milano-Bicocca, Milan, Italy

**Keywords:** Diagnosis, Signs and symptoms, Risk factors

## Abstract

Early research suggested that compulsive sexual behavior (CSB) and paraphilic interests (PI) are more prevalent in adults with primary tic disorders compared to the general population. However, recent data on this topic remain scarce. We conducted an anonymous online survey capturing data on CSB and PI in adult patients with primary tic disorders. We also explored the role of antipsychotic tic medication and the impact of neuropsychiatric comorbidities like attention-deficit hyperactivity disorder and depression. In total, 62 participants (26 females/36 males) completed the survey. The prevalence of CSB and PI were 12.9% and 19.4%, respectively. There was no association with antipsychotic medication nor with symptoms of depression. However, the presence of attention-deficit hyperactivity disorder was associated with a higher prevalence of both CSB and PI. The current results contrast with earlier reports and show that in adults with primary tic disorders, the prevalence of CSB and PI is not overly prominent.

## Introduction

Primary tic disorders, including chronic motor tic disorder (CMTD) and Tourette syndrome (TS) encompass childhood-onset neuropsychiatric disorders with the hallmark clinical feature of tics, but also an array of comorbid disorders. These most commonly include attention-deficit hyperactivity disorder (ADHD) and obsessive-compulsive disorder (OCD), as well as depression, anxiety, and sleep disorders [1]. Although primary tic disorders are most prevalent in childhood, in many cases both tics and associated conditions may also persist into adulthood and may be a cause of significant distress [2].

Early research into the structure of neuropsychiatric psychopathology of individuals with tic disorders linked further behavioral disorders to TS, including those affecting sexual health. For example, some clinical reports highlighted an elevated prevalence of paraphilic behaviors [3, 4] and clinically significant non-paraphilic compulsive sexual behaviors (CSB) in 32% of adolescents and adults with TS [5]. Other studies also explored associations of tics to CSB (also called “hypersexual behavior”) and paraphilias. One retrospective study of 358 adolescents and adults with TS demonstrated an increased prevalence of both CSB (in 25.7% of TS patients vs. 9.1% in controls) and paraphilic interests (PI) like fetishism (14.8% vs. 4.0%) [6], and also linked them to the presence of ADHD [6]. Severe sexual conduct disorders in adults with TS, including PI, were highlighted in several case reports [7–9]. The impact of antipsychotics was specifically emphasized in ameliorating paraphilic behavior like exhibitionism in one case and inappropriate sexual touching in another [7, 10], further drawing pathophysiological parallels between tic disorders and sexual disorders. Moreover, the presence of coprophenomena, i.e., the unintended expression of obscene actions (copropraxia) and/or utterances (coprolalia), was also associated with inappropriate sexual behaviors, although the exact behaviors were not specified [11]. Taken together, these studies arguably contributed to the notion that CSB and PI may be an endophenotype of some individuals with primary tic disorders including TS.

However, despite the early interest in sexual behaviors of people with primary tics described above, more recent data on this topic, specifically CSB and PI, are lacking. Given the detrimental social consequences that these behaviors may have for affected individuals and those around them in the absence of appropriate support, we conducted an online study to assess the prevalence of CSB and PI in adults diagnosed with a primary tic disorder. We also addressed the effects of antipsychotic medication on CSB and PI, and explored associations with two common comorbid conditions, namely ADHD and depression. Our goal is to inform the field, including medical and allied health professionals, but also people with tic disorders, on these sensitive but important issues, in order to provide appropriate guidance and help where needed.

## Methods

### Procedure

Adult patients from specialist tic disorders clinics with a confirmed diagnosis of a primary tic disorder (TS, chronic vocal tic disorder or CMTD) according to DSM 5 criteria [12] were asked whether they would be interested to participate in an online study conducted by the Department of Neurology with Experimental Neurology, at the Charité – Berlin University of Medicine related to sexuality and sexual health. Participants were informed about the study through three centers in Germany (Charité – Berlin University of Medicine, Hannover Medical School and University Medical Center Schleswig-Holstein) and one center in the UK (Birmingham and Solihull Mental Health NHS Foundation Trust). Interested individuals were then offered additional information materials that were provided by the study site (Charité – Berlin University of Medicine), including purpose, content and length of the study, as well as information about the study team. They were also provided with unique online access codes to a REDCap web-based software platform in a blinded manner that were generated by the Charité research team. Those individuals, who accessed the online study platform, were then offered a more detailed version of the aforementioned information and could anonymously participate by providing their consent clicking on a designated checkbox. Study participation was voluntary and without incentives.

The study consisted of a survey that was conducted either in the English or German language. The total data collected were part of a larger study that examined sexual health in individuals with primary tic disorders and comprised a total of 181 questions (unpublished data) presented on 25 pages. These encompassed demographic data (age, sex, gender, sexual orientation, diagnoses—including psychiatric comorbidities—medication, and relationship status), 64 questions on information specific to tic disorders, and 117 adaptive questions on sexuality and sexual health extracted from the German Health and Sexuality Survey (GeSiD) [13–15]. Upon survey completion, the participants could not revise their answers. The study included surveys with ≥80% of questions answered. The current paper focuses on the specific topics of CSB and PI. Participation was open for a period of 24 months between May 2020 and April 2022. Study data were collected and managed using REDCap web-based software platform [16] hosted at Charité University Hospital. All information materials, including the host server of REDCap, were sourced from Charité. All study documents and communications were handled by Charité. The online study did not contain personally identifiable information. The investigators involved in data handling were blinded to the code distribution process. That enabled to grant access to participants with specialist diagnosed primary tic disorders while maintaining anonymity in the study. Additional details about the study methodology according to the CHERRIES (Checklist for Reporting Results of Internet E-Surveys [17]); are provided in Supplementary Table 1.

### Participants

In total 69 participants partook the survey. After the exclusion of incomplete surveys (<80% filled out), 62 datasets remained. Sample characteristics are shown in Table 1. Table 2 provides a breakdown of the different drug classes prescribed to participants.

**Table 1:** Sample characteristics, frequency of primary tic disorder, comorbidities, medication, and current symptom severity in self-report scales for the total sample (*n* = 62).

Characteristic	Description	Number of participants (%)
Age (mean ± SD, years)	32.1 ± 10.3	
Sex	Male Female	36 (58.1%) 26 (41.9%)
Gender identity	Men Women Transgender Non-binary	36 (58.1%) 23 (37.1%) 2 (3.2%) 1 (1.6%)
Sexual orientation^a^	Solely heterosexual Mostly heterosexual Bisexual Homosexual Asexual	30 (53.6%) 13 (23.2%) 8 (14.3%) 4 (7.1%) 1 (1.8%)
Type of primary tic disorder	Tourette syndrome Chronic motor tic disorder	46 (74.2%) 16 (25.8%)
Previously diagnosed comorbidities^b^	Depression OCD Anxiety disorder ADHD Other undisclosed disorder None	20 (32.3%) 18 (29.0%) 12 (19.4%) 10 (16.1%) 4 (6.5%) 2 (3.2%)
Medication	Psychoactive medication use	35 (56.5%)
BDI-II (median, IQR (range))	11.5, 5.8–18.3 (0–37)	
ASRS Part A^c^ (median, IQR (range))	3.0, 1.5–4.0 (0–6)	
Current symptoms suggestive for ADHD^d^		17 (37.8%)

*SD* standard deviation, *OCD* obsessive-compulsive disorder, *ADHD* attention-deficit hyperactivity disorder, *IQR* interquartile range, *BDI-II* Beck Depression Inventory–II, *ASRS* ADHD Self-Report Scale.

^a^Data available from *n* = 56 participants.

^b^15 participants reported having received more than 1 diagnosis.

^c^Data available from *n* = 45 participants.

^d^Score above cutoff ≥4 in ASRS Part A.

**Table 2:** Frequency of reported psychoactive medication use, breakdown of medication classes.

	Total number
Psychoactive medication use^a^	35
Antipsychotics	16
Aripiprazole	10
Tiapride	2
Risperidone	1
Olanzapine	1
Pimozide	1
Amisulpiride	1
Other anti-tic medication	4
Topiramate	3
Tetrabenazin	1
Antidepressants	16
SSRI	13
Venlafaxin	1
Bupropion	1
Amitriptyline	1
Anxiolytic medication (Prothipendyl)	1
Cannabinoids	3
Amphetamine derivatives (Lisdexamfetamine)	1

*SSRI* selective serotonin reuptake inhibitor.

^a^Including data from participants taking multiple medications.

### Measures

Gender identity was captured as either male, female, trans*/transgender/transsexual or other (non-binary). For the purpose of this study, we used the data that captured CSB using the Hypersexual Behavior Inventory (HBI [18]); For German-speaking participants, a validated German HBI version was applied [19]. The HBI is a 19-item scale that assesses CSB on a 5-point Likert scale (1 = never to 5= very often), has a maximum score of 95 points and a cutoff at 53 points, which is suggestive of hypersexual symptoms of clinical significance. For PI, questions derived from ICD-11 [20] were used (see Supplementary Material). Questions explored sexual arousal patterns that focus on non-consenting others and are associated with substantial distress and/or risk of harm. The impact of ADHD and depression was explored using the World Health Organization Adult ADHD Self-Report Scale (ASRS) Part A (score ranging from 0–6, with a cutoff score ≥4 indicating current clinically significant ADHD) [21] and the BDI-II (score ranging from 0–63) [22], respectively.

### Statistical analyses

Data analysis was performed with SPSS Version 24.0 [23]. Demographic variables were presented as mean and standard deviation (SD); for non-normally distributed data, median and interquartile range (IQR, reported Q1–Q3) were calculated. The Shapiro–Wilk test of normality was applied for continuous data, like HBI, BDI-II and ASRS scores, which were all non-normally distributed. Categorical variables were compared between groups using the *χ*^2^ test (Fisher’s exact test, due to the small sample size). The Mann–Whitney *U* test was used to compare data on an interval scale between the two groups. The Kruskal–Wallis test was applied in comparisons between three or more groups. Spearman’s rank correlation was applied for correlation analyses between continuous variables.

## Results

The median HBI score of all participants was 29.5 (IQR = 22.0–41.0; range 19–78). No significant differences between male and female sex were found (*n*_male_ = 36, *n*_female_ = 26, *U* = 339.0, *z* = −1.843, *p* = 0.065). Eight participants (12.9%) scored above the predefined cutoff of ≥53 points suggestive of CSB, all of them were of male sex (*p* = 0.016). There was no difference in HBI scores between participants receiving antipsychotic medication and those who did not (*n*_antipsychotics_ = 16, *n*_no_antipsychotics_ = 46, *U* = 348.0, *z* = −0.322, *p* = 0.747; also see Table 2). HBI scores did not differ between participants with a previous diagnosis of depression and those without (*n*_depression_yes_ = 20, *n*_depression_no_ = 42, *U* = 419.5, *z* = −0.008, *p* = 0.994), and did not correlate with current depressive symptoms captured by the BDI-II (ρ(60) = 0.235, *p* = 0.066). There was no association between HBI scores and a previous diagnosis of ADHD (*n*_ADHD_yes_ = 10, *n*_ADHD_no_ = 52, *U* = 221.0, *z* = −0.748, *p* = 0.455). However, participants with an ASRS score above the cutoff and therefore suggestive of current clinically significant symptoms of ADHD were more likely to have an HBI score above the cutoff (*p* = 0.039).

Results on the prevalence of PI are shown in Table 3. There was no relation between the presence of PI and sex (*p* = 0.746). With respect to gender identity, eight individuals who reported PI identified as men, two as women and two as transgender. Participants who reported PI mostly stated to be solely or mostly heterosexual. There was no association between the presence of PI and clinically significant CSB (HBI ≥ 53 points) (*p* = 0.177), intake of antipsychotic medication (*p* = 1.0), a previous diagnosis of depression (*p* = 0.735), or BDI-II scores (*n*_paraphilias_yes_ = 12, *n*_paraphilias_no_ = 50, *U* = 270, *z* = −0.535, *p* = 0.593). The presence of PI was associated with a previous diagnosis of ADHD (*p* = 0.018), but not with current clinically significant ADHD symptoms based on the self-reported ASRS (score ≥4 points, *p* = 0.143).

**Table 3:** Prevalence of paraphilic ideation in primary tic disorders.

	Overall prevalence *N* (% of total)	Male sex *N* (% of male participants)	Female sex *N* (% of female participants)
Presence of paraphilic ideation	12 (19.4%)	8 (22.2%)	4 (15.4%)
Voyeurism	8 (12.9%)	7 (19.4%)	1 (3.8%)
Sadism	6 (9.7%)	3 (8.3%)	3 (11.5%)
Frotteurism	3 (4.8%)	1 (2.8%)	2 (7.7%)
Exhibitionism	2 (3.2%)	2 (5.6%)	–
Pedophilia	1 (1.6%)	–	1 (3.8%)
Coercive paraphilia	–	–	–
Distress through paraphilic ideation	4 (33.3%^a^)	2 (25%^b^)	2 (50%^c^)
Acted on paraphilic thoughts	1 (8.3%^a^)	1 (12.5%^b^)	–

^a^% of participants with paraphilic ideation.

^b^% of males with paraphilic ideation.

^c^% of females with paraphilic ideation.

## Discussion

This study explored CSB and PI via an online survey of 62 adults with a specialist-confirmed primary tic disorder. Its results showed that the prevalence of both CSB and PI is comparable between our sample and existing studies performed in the general population. Previous studies showed a prevalence ranging from 5.7% to 27.9% for CSB depending on the sex and age of the study population and a prevalence of ~18% for PI [24–26]. These results stand in contrast to earlier reports in TS that described a much higher prevalence of CSB and PI [3, 5, 6].

In the current sample, we found no differences in the average scores of the HBI (33.90; SD ± 10.46) compared, for example, to the average of the German sample (33.7; SD ± 14.5) [19]. Eight participants with chronic primary tics (22.2% of males, 12.9% of total participants) in this study did score high on the HBI scale (≥53 points), meeting the criteria for clinically significant CSB. This is, however, within the range reported by previous community-based studies (e.g. 5.7–15.8%) [26–28], and lower than rates reported in specific population subgroups, such as male university students, where the prevalence of clinically significant CSB is as high as 27.9% [27].

We also investigated further associations of CSB with sex, use of antipsychotic medication, presence of depression and ADHD. Our findings revealed no significant relation between overall CSB scores and sex or antipsychotic medication. However, all participants who met the criteria of a clinically significant CSB (HBI ≥ 53 points) were male. This finding is, however, not specific to primary tic disorders, as it has consistently been shown that CSB is generally more common in males [29]. We also found an association between self-reported scores consistent with the presence of ADHD and CSB. Previous literature has shown that the prevalence of ADHD is higher in patients with CSB [30], which was also demonstrated for adults with TS and ADHD [6]. However, our results do not provide support to the previously introduced notion that the presence of tics alone is related to CSB [6].

We reported a prevalence of 19.4% (*n* = 12) for PI in our primary tic cohort. Among individuals with PI, 33.3% (*n* = 4) reported distress from PI and 8.3% (*n* = 1) reported having acted out on at least one PI. These results are well within the prevalence range of PI reported in large population studies (18–45.6%) [24, 25], falling within the lower end of that range. This might be explained by the type of information captured in this study, which focused on clinically significant PI. Thus, the PI that were captured were limited to persistent and intense ideation involving sexual arousal patterns with a focus on non-consenting others or associated with substantial distress to the person affected or direct risk of injury or death to others [20]. Importantly, our results diverge from earlier reports on this topic in the TS population. Two previous studies reported a prevalence of inappropriate sexual behavior in 33.3% (*n* = 5/15) [3] and 32% (*n* = 16/50) individuals with TS [5]. This discrepancy may be related to the definition of paraphilic content. For example, Moldofsky et al. classified "voyeurism, repeated homosexual encounters, indecent genital exposure, incest and sadism" all under socially inappropriate or unacceptable sexual disturbances [3], whereas Nee et al. did not provide a clear definition [5]. It also remains unclear whether PI reported in these studies refer to ideation or to behavioral engagement. Interestingly, both studies reported a high prevalence of coprolalia in more than 58% of their cases. These numbers are surprisingly higher than the common average observed in people with primary tic disorders [11] and point toward differences in the characteristics of the clinical samples. Of note, an increased prevalence of PI and corresponding behaviors, such as exhibitionism, transvestism, sadomasochism, fetishism, and pedophilia was reported in adolescents and adults with TS, also in association with ADHD symptoms [6]. Although our results did not reveal any significant effects of current symptoms consistent with the diagnosis of ADHD on PI, there was an association between a preexisting diagnosis of ADHD and the presence of PI. This is in line with the results of a meta-analysis showing that the prevalence of ADHD is significantly higher in patients with PI compared to the general population [30]. Finally, we did not find any effects of antipsychotic medication on PI.

One limitation of this study is the relatively small number of participants, albeit larger than most previously conducted studies on the topic [3, 5, 6], which the authors contribute to both the nature of research topic, as well as the specific recruitment process through personal attendance in specialist clinics during the COVID-19 pandemic. However, this type of recruitment ensured that all study participants did have a diagnosis of a primary tic disorder and not tics or tic-like behaviors due to other etiologies, such as functional neurological disorder [31, 32]. Noteworthy is also that the study participants here had chosen to seek medical care for their tic disorders and therefore cannot be considered as representative of the entire spectrum of adults with primary tics, which includes people with milder tics and fewer comorbid neuropsychiatric symptoms. Moreover, we did not collect self-reported measures on tic severity and tic phenomenology. Capturing objective data by validated clinician-applied scales like YGTSS (Leckman et al. [33]) would have been desirable but was not possible due to the anonymous nature of this study. Another limitation relates to the sex ratio of our participants, which had a higher proportion of female participants than what is typically described in other TS cohorts, specifically in childhood (1.3:1 vs. 4.3:1) [34]. However, it has been suggested that male predominance becomes less evident in adulthood [35], in line with our sample. The higher proportion of female participants in this study might have resulted in lower HBI scores, as CSB is more commonly observed in males [29]. Moreover, our study did not capture symptoms related to OCD, as we had to carefully balance the overall number of survey queries and the most relevant hypotheses pertaining to primary tic disorders.

## Conclusions

We did not find evidence for a high prevalence of CSB or PI in adults with a primary tic disorder. There was, however, a relevant association between comorbid ADHD and both CSB and PI, which should be considered in the diagnostic and therapeutic work-up of these patients.

## Supplementary information

Supplementary table

## Data Availability

The data that support the findings of this study are available from the corresponding author, LK, upon reasonable request.
