# Supplementary material for: Compulsive sexual behavior and paraphilic interests in adults with chronic tic disorders and Tourette syndrome: a survey-based study
Source: Int J Impot Res. 2023 Jul 19;37(3):233–7. doi: 10.1038/s41443-023-00729-x (PMC11981945; doi:10.1038/s41443-023-00729-x)
Supplement: Supplementary file 1 — Supplementary table [file 41443_2023_729_MOESM1_ESM.docx]

**Supplementary table 1**. Checklist for Reporting Results of Internet E-Surveys (CHERRIES)(adapted from Eysenbach 2004).

| ***Checklist Item*** | ***Location in paper*** | ***Description*** |
| --- | --- | --- |
| Survey design | Pages 4-6 | The target population of this study were adult patients with primary tic disorders (Tourette syndrome, chronic motor tic disorders, chronic vocal tic disorder). The goal of this particular study was to assess the presence of compulsive sexual behaviors and paraphilic interests in this clinical population largely using questions from validated instruments. |
| IRB approval | Page 16 | The study has been approved by the IRB of the Charité University Medicine Berlin, Germany, (EA2_086_19). |
| Informed consent | Pages 4-5 | Patients who attented clinics at the following institutions (Charité – Berlin University of Medicine, Hannover Medical School, University Medical Center Schleswig-Holstein and Birmingham and Solihull Mental Health NHS Foundation Trust) were informed on the possibility of this ongoing study conducted at the Charité – Berlin University of Medicine. Interested individuals were then provided with additional information materials offered by the study site (Charité – Berlin University of Medicine), including purpose, content and length of the study, as well as information about the study team. A link to a REDCap web-based software platform alongside with a unique online access code generated by the Charité research team were also provided. Those individuals, who accessed the online study platform with the provided code, were offered a more detailed version of the aforementioned information and where they could anonymously participate by providing their consent clicking on a designated checkbox. |
| Data protection | Page 5 | The REDCap web-based software platform we used has a database server located behind a firewall managed by the hosting insitutition (Charité). REDCap study databank is only accessible to the study investigators (Lille Kurvits, Tina Mainka, Christos Ganos) from the study site (Charité), who were blinded to clinical outpatient care. Participants were only able to access the study questionnaire using their unique access code. No personal identifying data was collected. The data gathered and stored in the REDCap databank did not contain any personal identifiers. Upon completing the study, the participants were not able to access the survey again. |
| Development and testing | Page 5 | The study uses items adapted from the GeSiD (population-based German health and sexuality survey) questionnaire. Additionally, items from validated scales regarding comorbid symptoms were added to the study. Usability and technical functionality of the electronic questionnaire were tested in house by the REDCap development team and the investigator team. |
| Open survey versus closed survey | Page 5 | This is a closed survey which each participant could access through the use of a unique access code and upon providing informed consent. The investigators involved in data handling were blinded to the process of code distribution. |
| Contact mode | Page 4-5 | Participants were informed about the study and its content by their respective healthcare providers. In case of further interest, they were provided with written information created by the local Charité research team, which also included a link and an access code for the online survey. |
| Advertising the survey | n/a | The study was not advertised outside the specialist clinic appointments. |
| Web/E-mail | n/a | This web-based study was hosted by the REDCap software platform and used an automatic method for capturing responses. |
| Context | n/a | The study did not use online recruitment methods. The sampling strategy involved recruiting specialist-diagnosed participants, who were handed unique access codes. |
| Mandatory/voluntary | Page 5 | Participation was voluntary. |
| Incentives | Page 5 | There were no incentives provided. |
| Time/Date | Page 5 | Participation was open for a period of 24 months between May 2020 and April 2022. |
| Randomization of items or questionnaires | n/a | Items in the study were not randomized or alternated. |
| Adaptive questioning | Page 5 | Adaptive questioning was used to reduce number and complexity of the questions. |
| Number of Items | Page 5 | This online study was organized into blocks consisting of topic-specific questions. In total the study comprised maximally 181 items. In the current publication only data regarding the topic at hand are presented and discussed. |
| Number of screens (pages) | Page 5 | The total study questionnaire consisted of 25 pages. |
| Completeness check | Page 5 | Completeness check was done after submission. Data were included in the final analysis if ≥ 80% of the relevant study questions were filled out. |
| Review step | Page 5 | Respondents were able to review and change their answers through a „back“ button before submission. Participants could also pause and return later using an access code the REDCap system generated for secure re-access of the study portal. Upon completing the survey, participants could not re-enter the study platform or revise their answers. |
| Unique site visitor | n/a | We did not determine unique site visitors based on IP addresses or cookies, as participation was only possible via unique access code. |
| View Rate | n/a | Not available, as there was no separate visitor online site. |
| Participation rate (Ratio of unique visitors who agreed to participate/unique first survey page visitors) | n/a | All 69 participants who accessed the study platform filled in at least the first survey page (by agreeing to participate by checking a checkbox of informed consent). There was no information captured on how many information brochures/codes were overall shared with interested individuals. |
| Completion rate (Ratio of users who finished the survey/users who agreed to participate) | Not referenced in the main body | In total 62 participants completed the study (i.e. ≥ 80% of the study was filled out) which yields a completion rate of 89.9 %. |
| Cookies used | n/a | REDCap platform uses cookies to store information about the user's session, such as their login credentials and preferences. However, these cookies are not used to assign a unique user identifier to each client computer. Instead of relying on cookies, REDCap uses other methods to assign a unique user identifier to each user. For example, users were required to enter a unique password when logging into the platform, which provides a reliable and secure means of identifying individual users. |
| IP check | n/a | While the REDCap platform does capture the IP address of the client computer when a user logs in or performs any action, this information is not used by REDCap to identify duplicate entries, but as a rate limiter of too many requests from the same IP adress (limit at 600 requests). Instead of relying on IP addresses, REDCap provides a number of built-in tools and features to help researchers identify and manage duplicate entries. This study used unique identifiers for each participant, which were used to prevent duplicate entries. |
| Log file analysis | n/a | REDCap does not have any built-in techniques for automatically identifying such multiple entries. Instead, the log files were reviewed manually to identify any instances of multiple entries. This involved comparing the date and time stamps of the entries and looking for duplicate entries or inconsistencies. |
| Registration | n/a | This survey is a “closed” (non-open) survey where participants needed to first login using a unique access code which prevented duplicate entries from the same participant. Participants could pause the study and re-enter the questionnaire using platform-generated unique re-entry code. Once the study questionnaire was submitted, it was not possible to access the database again. |
| Handling of incomplete questionnaires | Not referenced in the main body | Incomplete entries were registered and stored in the database as well included in the final analysis if ≥ 80% of the study was filled out. Entries missing > 20 % of data were discarded from the final analysis (i.e., 7 entries). |
| Questionnaires submitted with an atypical timestamp | Not referenced in the main body | No specific timeframe or cut-off points were used. |
| Statistical correction | Not referenced in the main body | No extra correction analysis such as weighting of items or propensity scores was done to adjust the data. |
